# Publisher Correction: Cytoplasmic FBXO38 mediates PD-1 degradation

**DOI:** 10.1038/s44319-024-00298-0

**Published:** 2024-11-01

**Authors:** Xiwei Liu, Xiangbo Meng, Zuomiao Lin, Shutan Jiang, Haifeng Liu, Shao-cong Sun, Xiaolong Liu, Penghui Zhou, Xiaowu Huang, Lai Wei, Wei Yang, Chenqi Xu

**Affiliations:** 1 CAS Center for Excellence in Molecular Cell Science, Shanghai Institute of Biochemistry and Cell Biology, Chinese Academy of Sciences, Shanghai, China; 2 Advanced Medical Research Institute, Meili Lake Translational Research Park, Cheeloo College of Medicine, Shandong University, Jinan, Shandong China; 3 School of Life Science, Hangzhou Institute for Advanced Study, University of Chinese Academy of Sciences, Hangzhou, China; 4 Institute of Immunology, Chinese Institutes for Medical Research, Beijing, China; 5 State Key Laboratory of Oncology in South China, Collaborative Innovation Center for Cancer Medicine, Sun Yat-sen University Cancer Center, Guangzhou, China; 6 Department of Liver Surgery and Transplantation, Liver Cancer Institute, Zhongshan Hospital, Fudan University, Shanghai, 200032 China; 7 Key Laboratory of Carcinogenesis and Cancer Invasion (Fudan University), Ministry of Education, Shanghai, 200032 China; 8 Shanghai Key Laboratory of Organ Transplantation, Zhongshan Hospital, Fudan University, Shanghai, 200032 China; 9 Guangdong Provincial Key Laboratory of Allergy & Clinical Immunology, The Second Affiliated Hospital, Guangzhou Medical University, Guangzhou, 510000 China; 10 Guangdong Provincial Key Laboratory of Molecular Oncologic Pathology, Department of Pathology, School of Basic Medical Sciences, Southern Medical University, Guangzhou, China; 11 Department of Pathology, Nanfang Hospital, Southern Medical University, Guangzhou, China

## Abstract

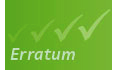

**Correction to:** *EMBO Reports* (2024) 25:4168–4171. 10.1038/s44319-024-00254-y | Published online 16 September 2024

Callouts were updated during the publication process of the referenced paper, N Dibus et al - *EMBO Rep* (2024) 25:4206–4225 10.1038/s44319-024-00220-8.

**The figure callout out to EV2 on page 3 of the manuscript is corrected**.

Figure callout is updated from:

Fig. EV2 of Dibus et al has several caveats:

To: (Change in bold)

Fig. **EV3** of Dibus et al has several caveats:

The original article has been corrected.

