# Supplementary material for: Cytoplasmic FBXO38 mediates PD-1 degradation
Source: EMBO Rep. 2024 Sep 16;25(10):4168–71. doi: 10.1038/s44319-024-00254-y (PMC11467372; doi:10.1038/s44319-024-00254-y)
Supplement: Supplementary file 2 — Appendix [file 44319_2024_254_MOESM2_ESM.pdf]

Materials and Methods

Reagents

Flow cytometry antibodies, including anti-mCD4-PE (RM4-5), anti-mFoxp3-eFluor 450 (FJK-16s), and anti-mPD-1-APC (RMP1-30), were purchased from Thermo Fisher. For western blotting, anti-FBXO38 (ab87729) was from Abcam; anti-GAPDH (Cat#KC-5G5) was from Aksomics; anti-Lamin B1 (Cat#12987-1-AP) was from Proteintech; and anti-HA (3F10) was from Roche. For imaging experiments, anti-GM130 (Cat#610823) and anti-EEA1 (Cat#610457) were from BD; anti-PD1 (ab216352), anti-TGN46 (ab2809) and anti-CANX (ab22595) were from Abcam; anti-RAB11 (Cat#71-5300) and anti-TFR (H68.4) were from Thermo Fisher. For PLA experiment, anti-HA tag (6E2) was from Cell Signaling Technology; anti-PD-1 (CAL20) was from Abam; Duolink® In Situ Red Starter Kit (Cat#DUO92101) was from Merck.

Constructs

Human *FBXO38* transcript isoforms were amplified from Jurkat cell cDNA and subsequently cloned into pHAGE expression vectors with 3×Myc tag for ectopic expression in Jurkat as previously described (Meng *et al*, 2018). All constructs were confirmed by sequencing.

Cells

HEK293 and Jurkat cells were purchased from the Cell Bank of Chinese Academy of Sciences (Shanghai, China). Jurkat cells with *FBXO38-HA* knockin and *FBXO38* knockout were described previously (Meng *et al.*, 2018). All cell lines were tested negative for mycoplasma contamination. HEK293 cells were cultured with complete DMEM medium containing 10% FBS and antibiotics (100 µg/ml streptomycin and 100 units/ml penicillin, GIBCO). Jurkat cells were cultured in RPMI-1640 medium containing 10% FBS and antibiotics. All cells were cultured at 37 °C in an atmosphere of 5% CO_2.

Nucleus-cytoplasm fractionation

The method is modified from a previous publication (Rosner *et al*, 2007). Eight million Jurkat cells were harvested and washed twice gently with PBS in a 1.5 ml Eppendorf tube. The cells were then resuspended in 200 µl buffer F1 (20 mM Tris, 50 mM 2-mercaptoethanol, 0.1 mM EDTA, 2 mM $MgCl_2$, and a protease inhibitor cocktail, with final pH adjusted to 7.5-7.6) and incubated for 2 min at room temperature followed by ice incubation for 10 minutes. NP-40 was added to a final concentration of 0.1%, and the cells were then passed through a 0.7 mm syringe needle at least three times. Cell lysates were centrifuged at 800g for 5 min at 4 °C. The supernatant was transferred to

a new tube and centrifuged at 21,000g with a fixed-angel rotor for 15 min at 4 °C to remove debris. Another round of centrifugation was performed with a swinging bucket rotor at 3000g for 10 min at 4 °C to further remove debris, thus generating the cytoplasmic fraction. The pellet was washed three times with buffer F1 containing 0.1% NP-40 without resuspending or vortex to get the nuclear fraction. Both the cytoplasmic and nuclear fractions were boiled with loading buffer for 15 minutes, followed by centrifugation before gel electrophoresis and western blotting analysis.

Confocal imaging

WT or *FBXO38-HA* knockin Jurkat cells were stimulated with PHA (150 ng/ml) for 72h to induce PD-1 expression. The cells were loaded on poly-Lysine coated dish, fixed with IC Fixation Buffer (Thermo Fisher, 00-8222-49) for 20 min at room temperature and stained with unconjugated primary antibodies in permeabilization buffer (Thermo Fisher, 00-8333-56) for 10 h at 4°C. Remove the primary antibody solutions and wash the samples 2 x 5 min, then cells were stained with AF488 or AF647 conjugated secondary antibodies for 1 h and DAPI for 5 min at room temperature. Images were collected by Leica SP8 confocal microscope.

Duolink® PLA fluorescence analysis

For the interaction between endogenous FBXO38 and PD-1, WT or *FBXO38-HA* knockin Jurkat cells were stimulated with PHA (150 ng/ml) for three days to induce PD-1 expression. The samples were fixed with IC Fixation Buffer (Thermo Fisher, 00-8222-49) for 20 min at room temperature and permeabilized with permeabilization buffer (Thermo Fisher, 00-8333-56) for 30 min at room temperature. The proximity ligation assay was performed according to the manufacture protocol (Merck, Cat#DUO92101), using the primary antibodies anti-HA (for FBXO38) and anti-PD-1. WT Jurkat cells were negative control. Images were collected by Leica SP8 confocal microscope. The PLA signals were quantified with ImageJ software.

Mice and ethics statement

$Fbxo38^{flox}$ mice were generated as described in the previous publication (Meng *et al.*, 2018). All mice were C57BL/6 background. $Fbxo38^{flox/flox}$ mice were crossed with $Foxp3^{YFP\text{-}Cre}$ mice (from The Jackson Laboratory) to get $Fbxo38^{Treg\text{-}CKO}$ mice with FBXO38 deficiency in Treg cells. Animal experiments involving $Fbxo38^{Treg\text{-}CKO}$ mice were controlled using littermates with normal FBXO38 expression. All animal experiments used male mice matched for age (18-month). All mice were maintained in pathogen-free facilities in the Animal Resource Center at Shandong University, Jinan, Shandong Province, China.

All the animal experiments involved were approved by the Animal Care and Animal Experiments Committee of Shandong University (ECSBMSSDU2020-2-054) and complied with all relevant ethical regulations.

PD-1 surface levels of Treg cells

To measure PD-1 expression in splenic Treg cells, splenocytes were stained with anti-PD-1 and anti-CD4 antibodies and washed with PBS for 2 times, then fixed, permeabilized and stained with anti-Foxp3 antibody with Transcription Factor Staining Buffer Set (Catalog: 00-5223-56, eBioscience) according to the manufacturer's protocol. Stained cells were washed 2 times with PBS prior to Flow Cytometry analysis. Treg cells were gated as $CD4^+$ $Foxp3^+$ cells, and isotype control IgG was used to gate PD-1 positive cells.

Statistical analysis

Statistical analyses were performed using GraphPad Prism 8 (GraphPad Software, Inc.). For the data presented in Fig. 1i and 1j, the distribution was first assessed using the Kolmogorov-Smirnov test. Data in Fig. 1i did not conform to a normal distribution, and therefore were analyzed by Mann–Whitney U-test. The data in Fig. 1j followed a normal distribution with comparable variances, allowing for the application of a two-tailed unpaired Student's t-test. The figures display mean values with error bars representing ± SEM. Statistical significance was represented as *$p< 0.05$ and **** $P < 0.0001$.

Data availability

No data have been deposited in public databases.

All original western blots, immunofluorescence images and quantitative values are available as source data associated with the Figure 1.

Reference

Meng X, Liu X, Guo X, Jiang S, Chen T, Hu Z, Liu H, Bai Y, Xue M, Hu R *et al* (2018) FBXO38 mediates PD-1 ubiquitination and regulates anti-tumour immunity of T cells. *Nature* 564: 130-135

Rosner M, Hanneder M, Freilinger A, Hengstschlager M (2007) Nuclear/cytoplasmic localization of Akt activity in the cell cycle. *Amino Acids* 32: 341-345
