# Supplementary figures and images for: Cytoplasmic FBXO38 mediates PD-1 degradation
Source: EMBO Rep. 2024 Sep 16;25(10):4168–71. doi: 10.1038/s44319-024-00254-y (PMC11467372; doi:10.1038/s44319-024-00254-y)

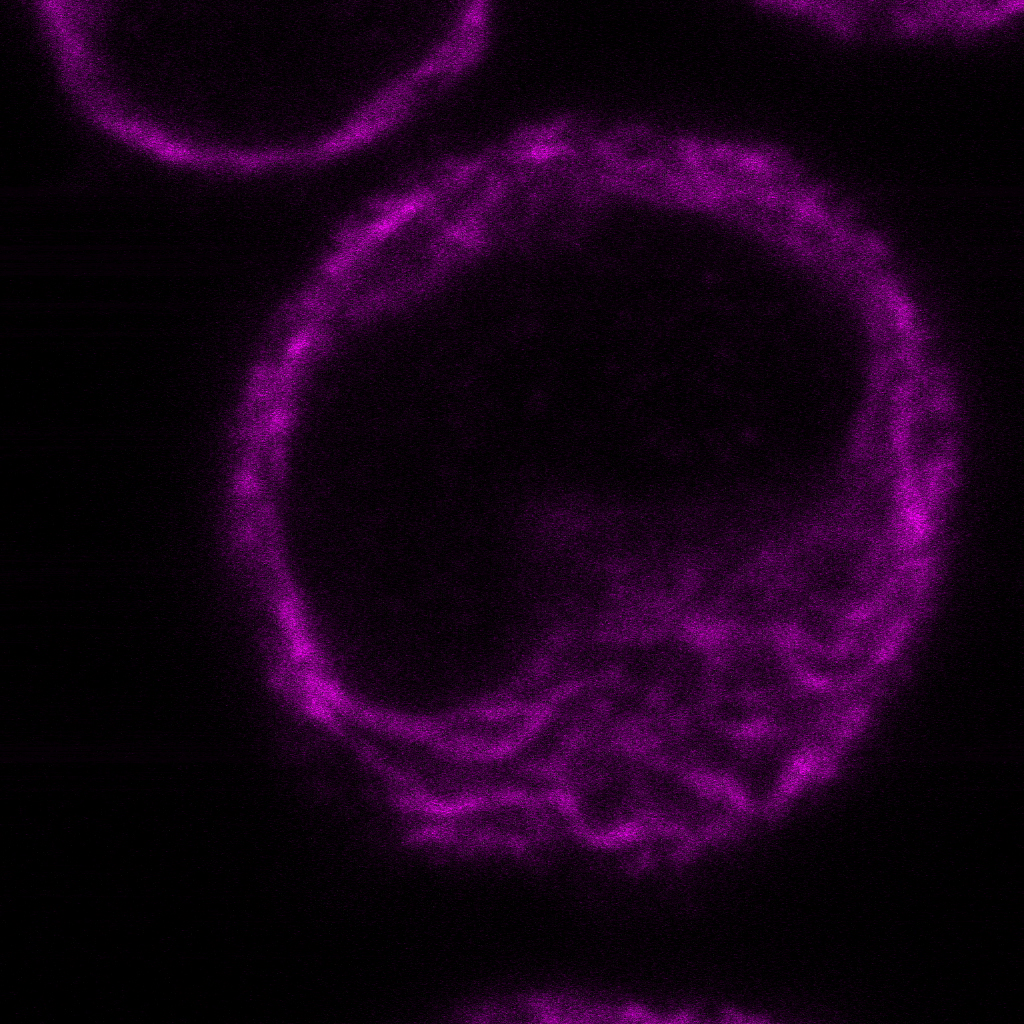

Supplement: Supplementary file 3 — Source data Fig. 1 [file 44319_2024_254_MOESM3_ESM.zip › Source data for Fig. 1h/Fig. 1h/CANX_CANX.tif]

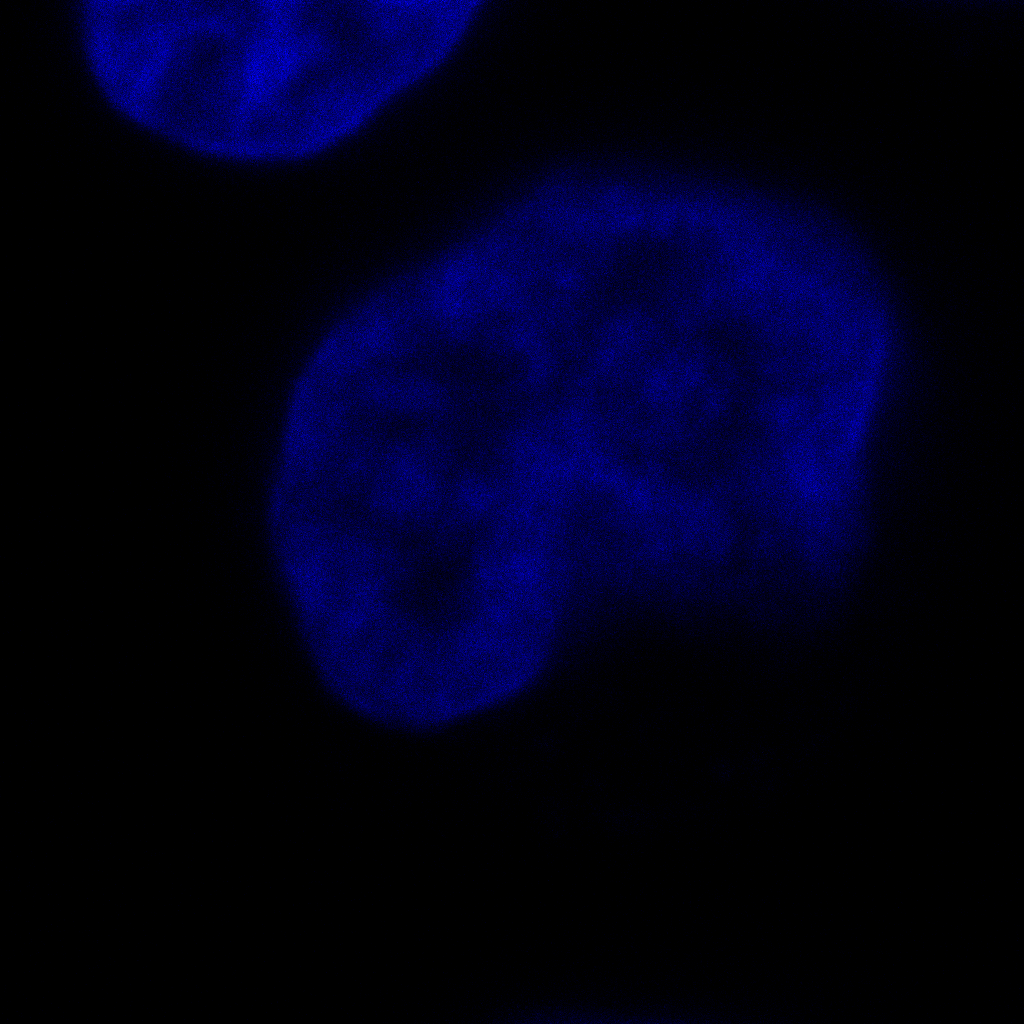

Supplement: Supplementary file 3 — Source data Fig. 1 [file 44319_2024_254_MOESM3_ESM.zip › Source data for Fig. 1h/Fig. 1h/CANX_DAPI.tif]

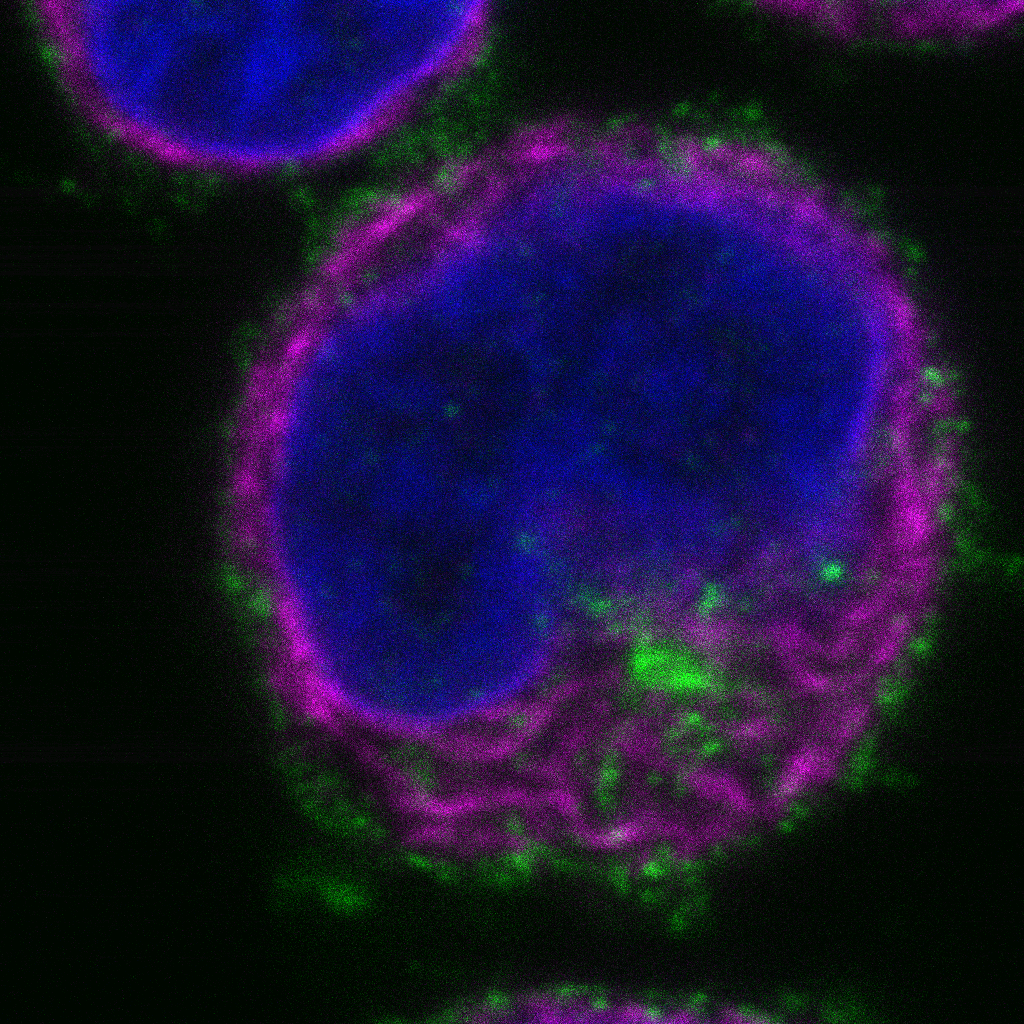

Supplement: Supplementary file 3 — Source data Fig. 1 [file 44319_2024_254_MOESM3_ESM.zip › Source data for Fig. 1h/Fig. 1h/CANX_Merge.tif]

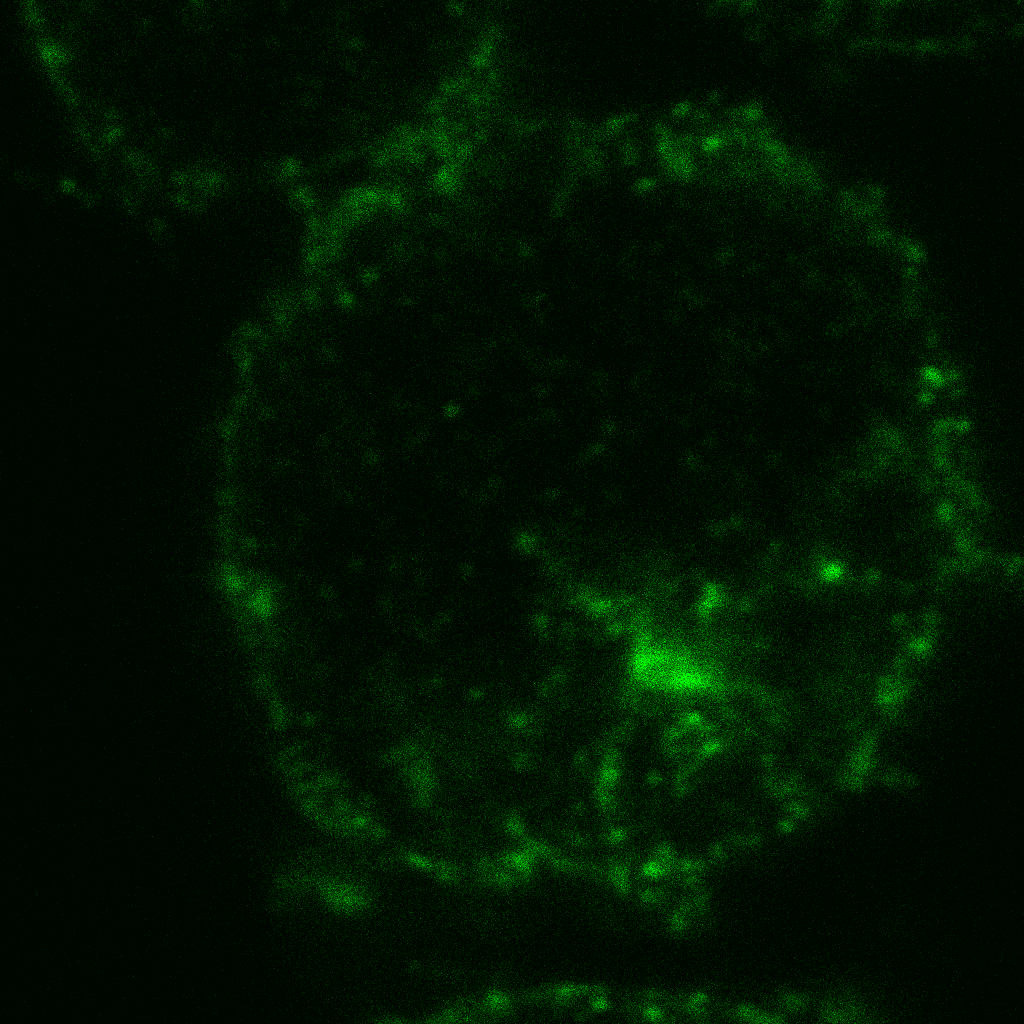

Supplement: Supplementary file 3 — Source data Fig. 1 [file 44319_2024_254_MOESM3_ESM.zip › Source data for Fig. 1h/Fig. 1h/CANX_PD-1.tif]

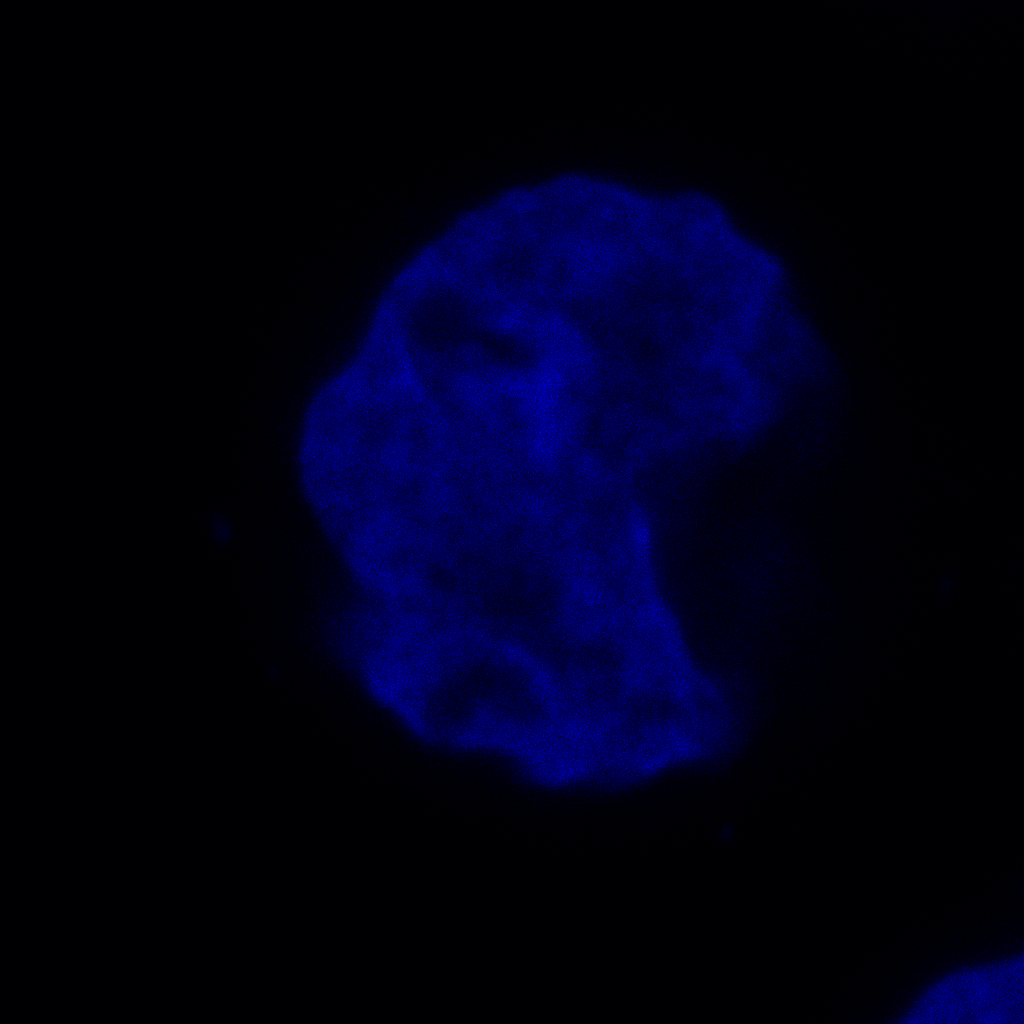

Supplement: Supplementary file 3 — Source data Fig. 1 [file 44319_2024_254_MOESM3_ESM.zip › Source data for Fig. 1h/Fig. 1h/EEA1_DAPI.tif]

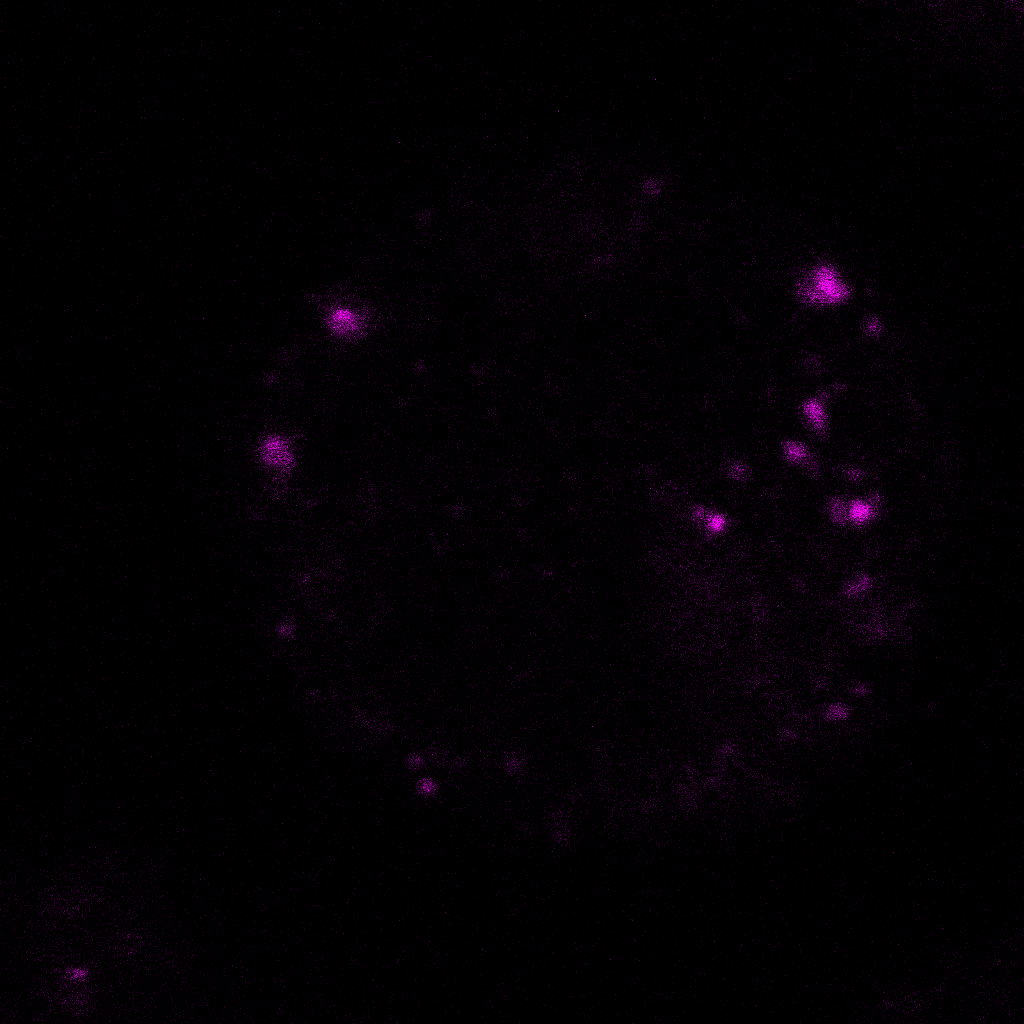

Supplement: Supplementary file 3 — Source data Fig. 1 [file 44319_2024_254_MOESM3_ESM.zip › Source data for Fig. 1h/Fig. 1h/EEA1_EEA1.tif]

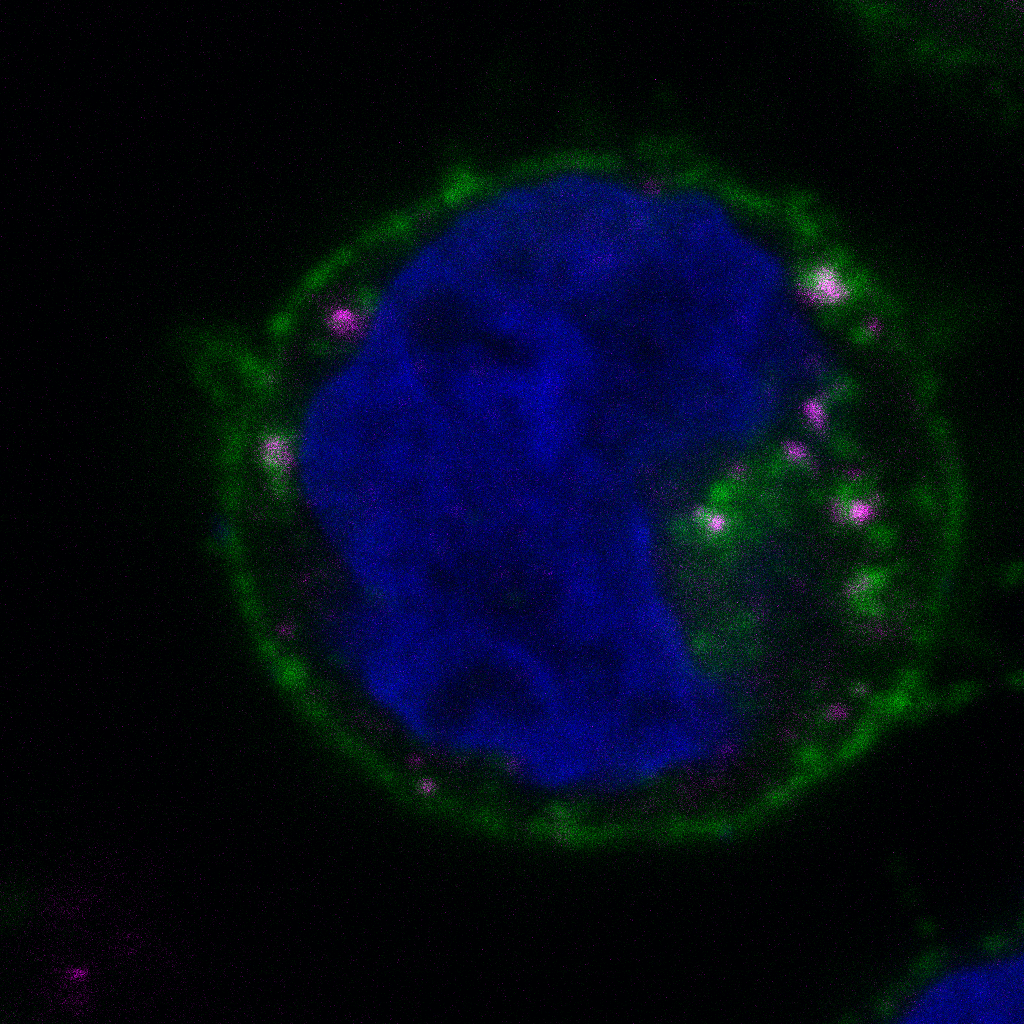

Supplement: Supplementary file 3 — Source data Fig. 1 [file 44319_2024_254_MOESM3_ESM.zip › Source data for Fig. 1h/Fig. 1h/EEA1_Merge.tif]

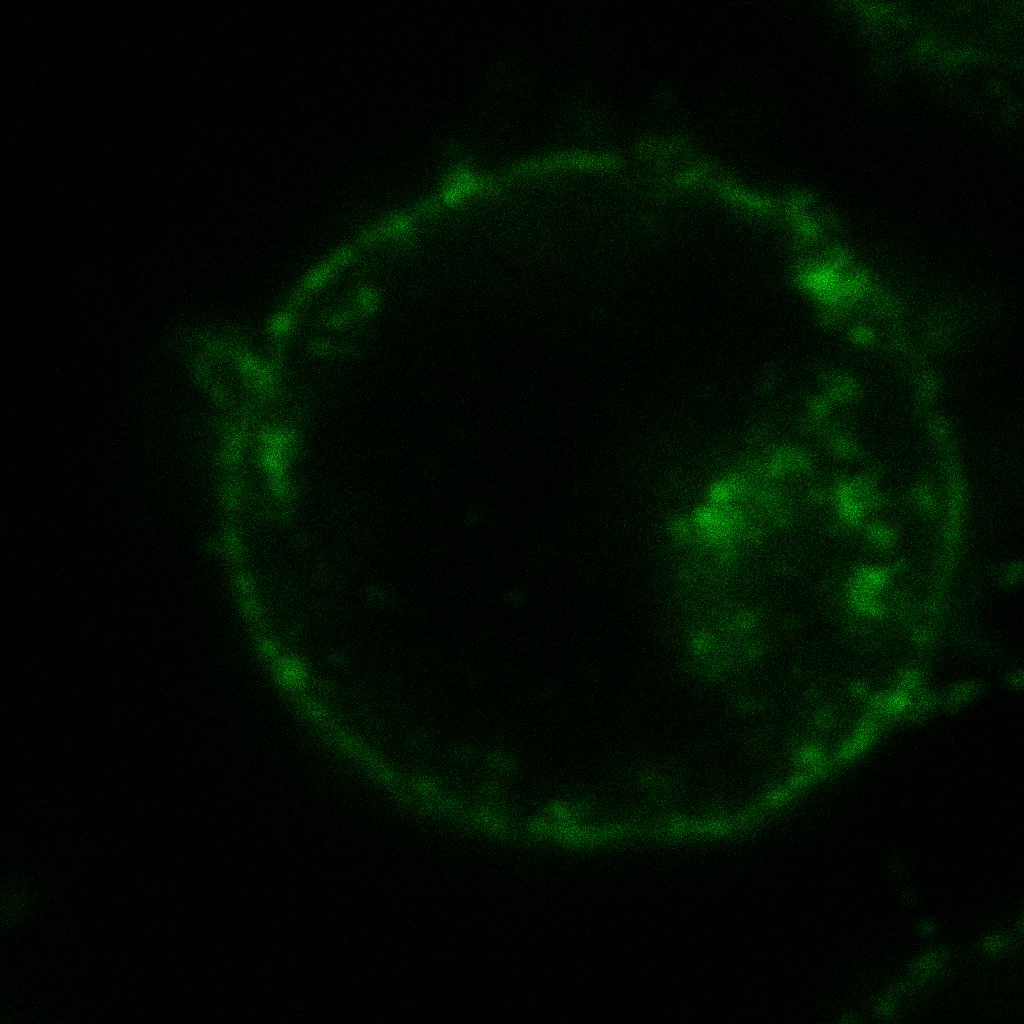

Supplement: Supplementary file 3 — Source data Fig. 1 [file 44319_2024_254_MOESM3_ESM.zip › Source data for Fig. 1h/Fig. 1h/EEA1_PD-1.tif]

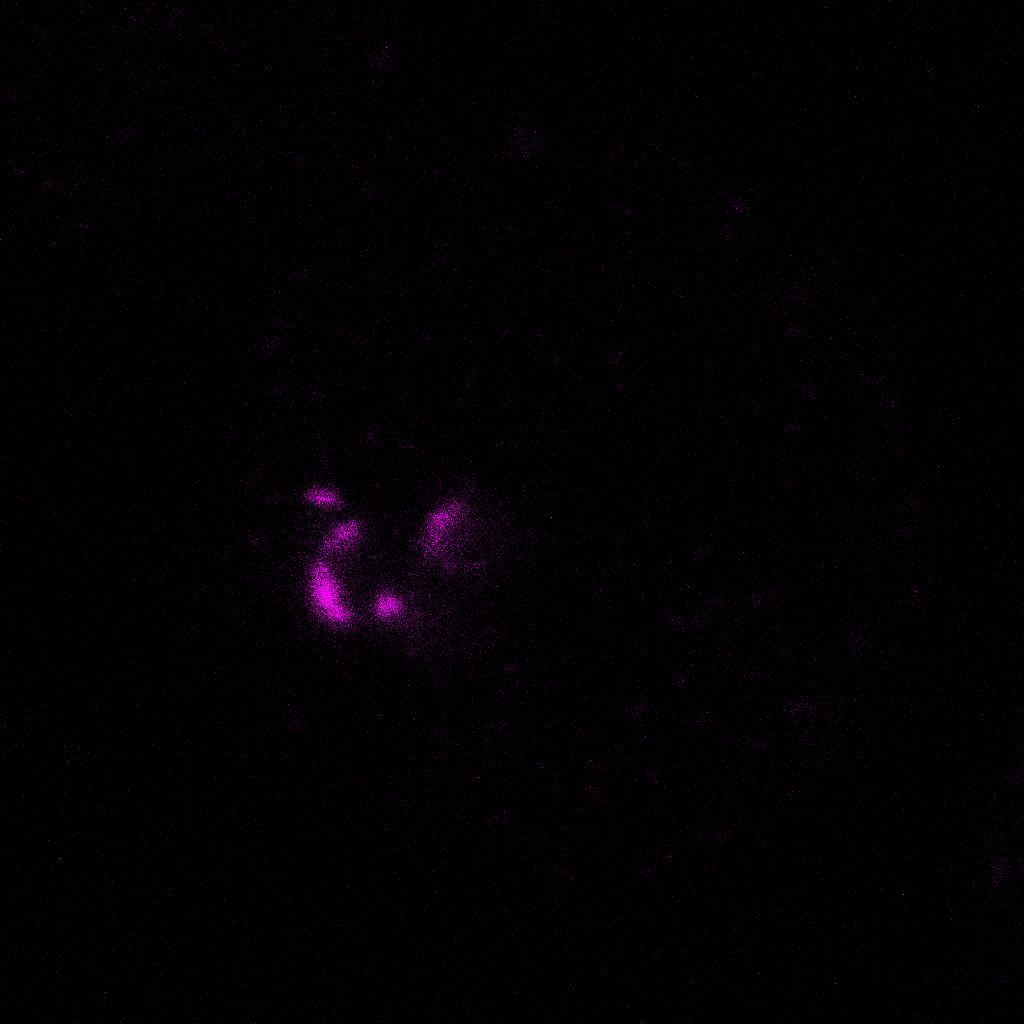

Supplement: Supplementary file 3 — Source data Fig. 1 [file 44319_2024_254_MOESM3_ESM.zip › Source data for Fig. 1h/Fig. 1h/GM130_CM130.tif]

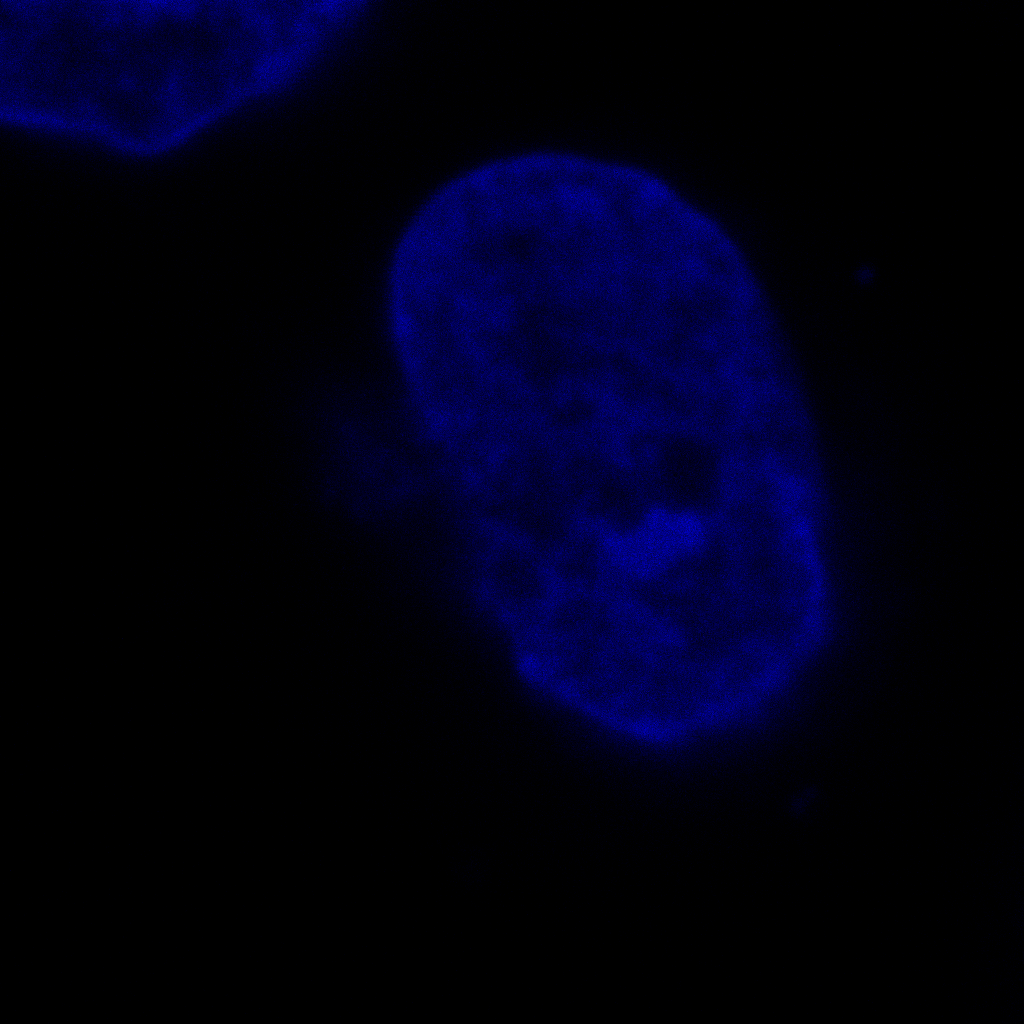

Supplement: Supplementary file 3 — Source data Fig. 1 [file 44319_2024_254_MOESM3_ESM.zip › Source data for Fig. 1h/Fig. 1h/GM130_DAPI.tif]

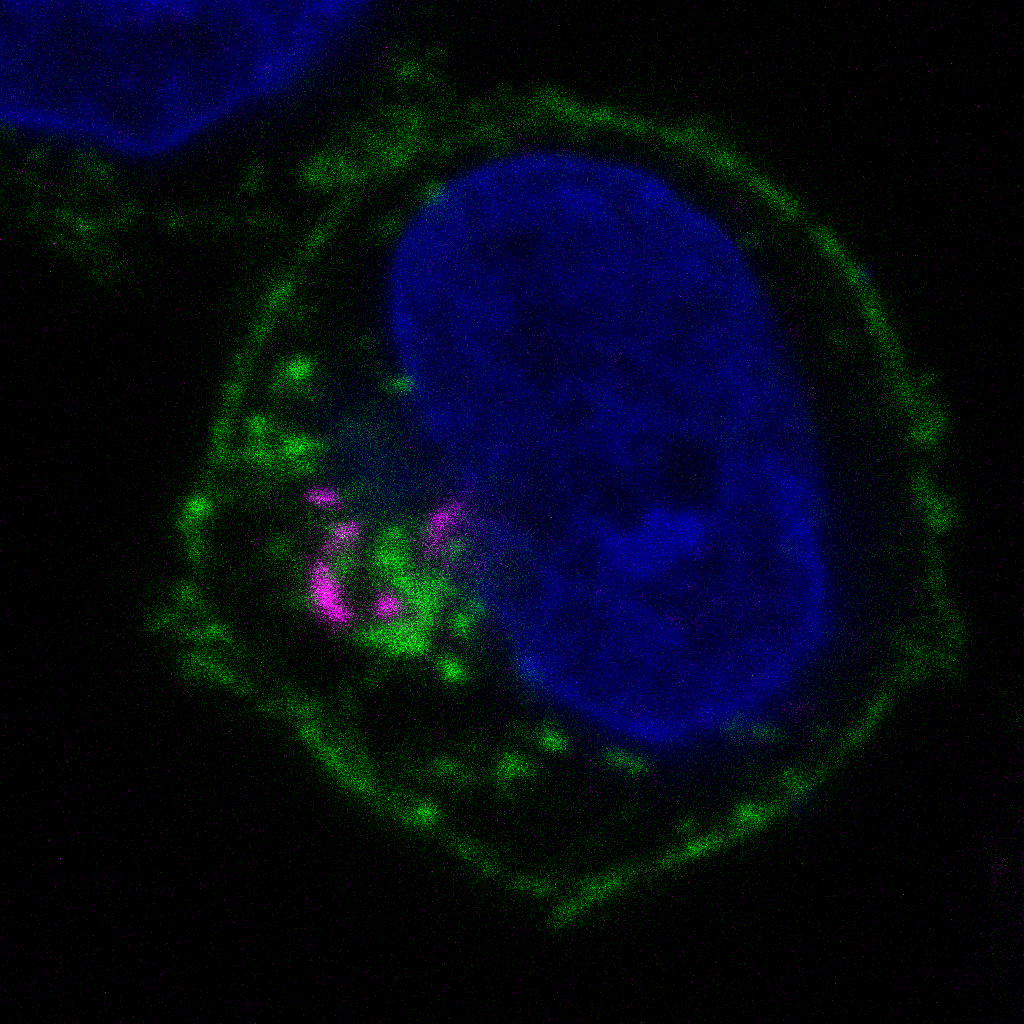

Supplement: Supplementary file 3 — Source data Fig. 1 [file 44319_2024_254_MOESM3_ESM.zip › Source data for Fig. 1h/Fig. 1h/GM130_Merge.tif]

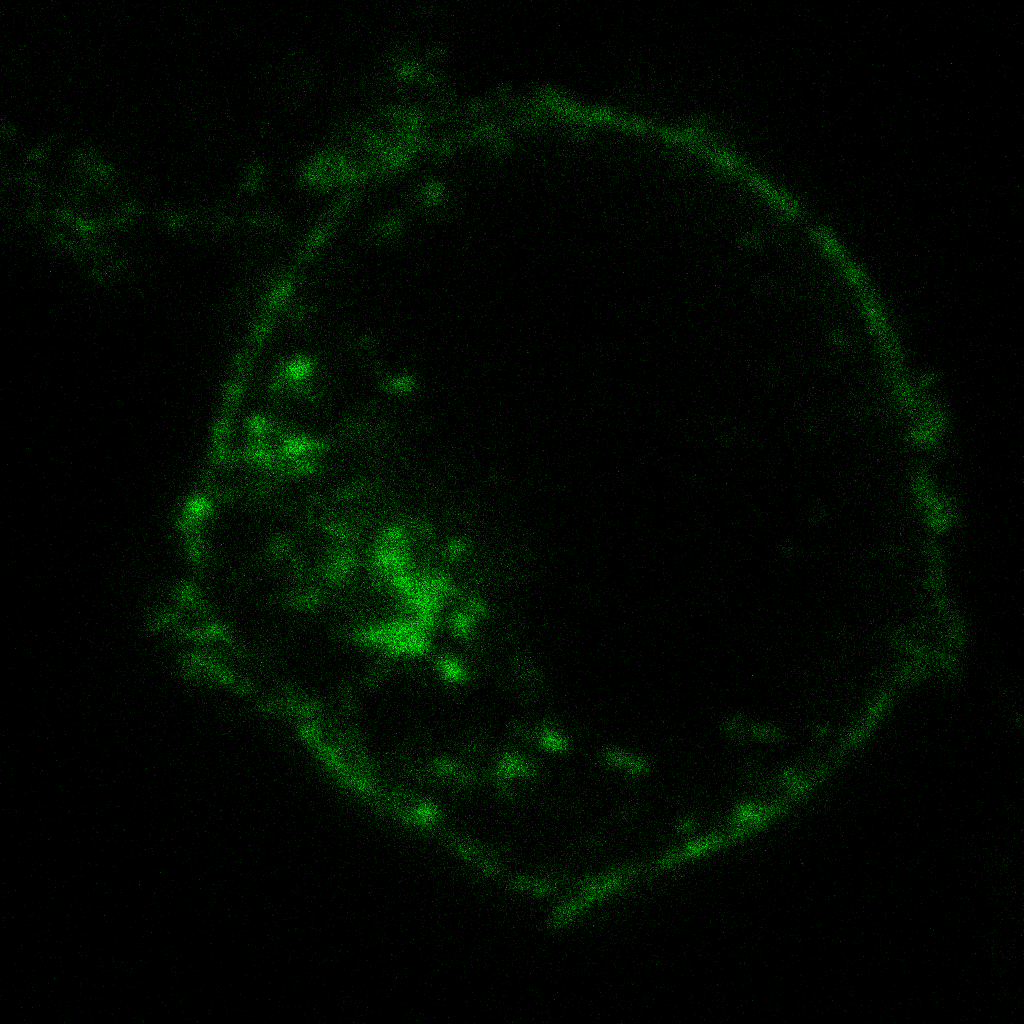

Supplement: Supplementary file 3 — Source data Fig. 1 [file 44319_2024_254_MOESM3_ESM.zip › Source data for Fig. 1h/Fig. 1h/GM130_PD-1.tif]

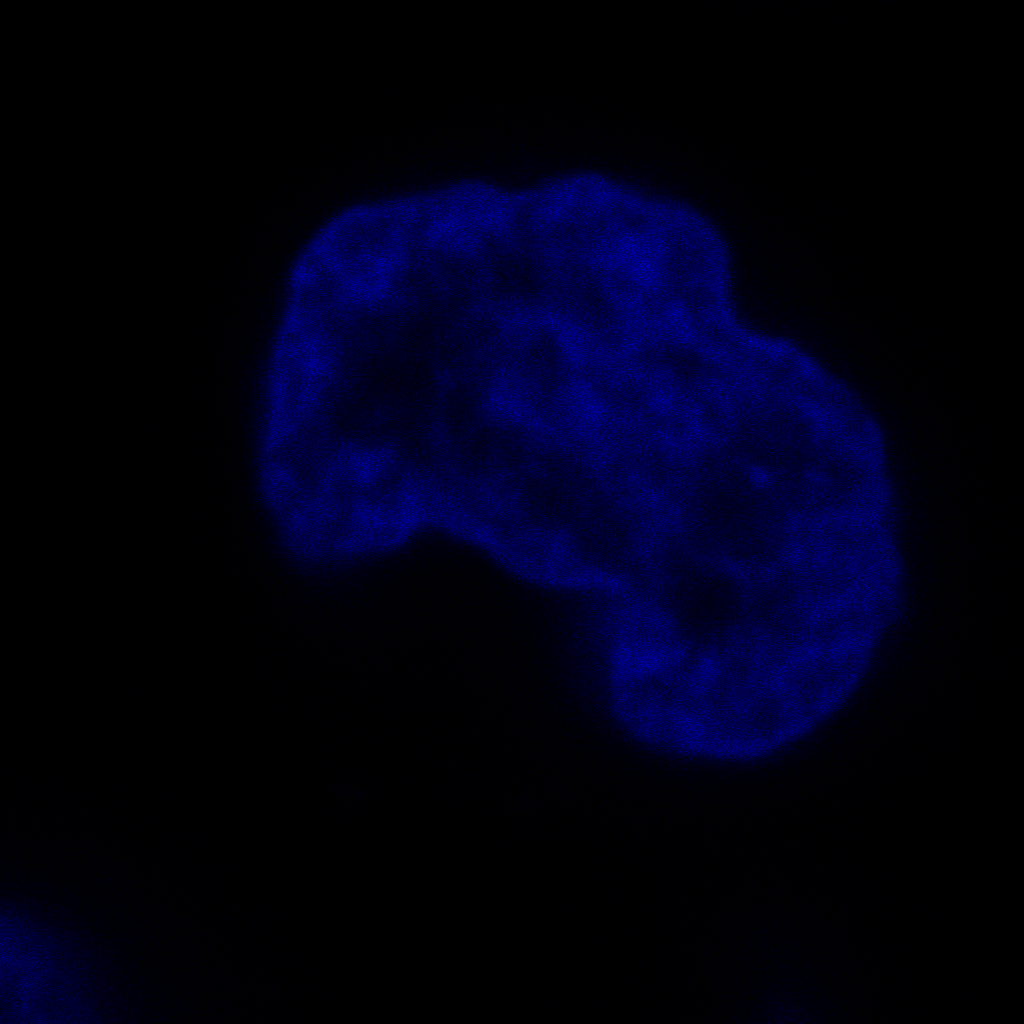

Supplement: Supplementary file 3 — Source data Fig. 1 [file 44319_2024_254_MOESM3_ESM.zip › Source data for Fig. 1h/Fig. 1h/RAB11_DAPI.tif]

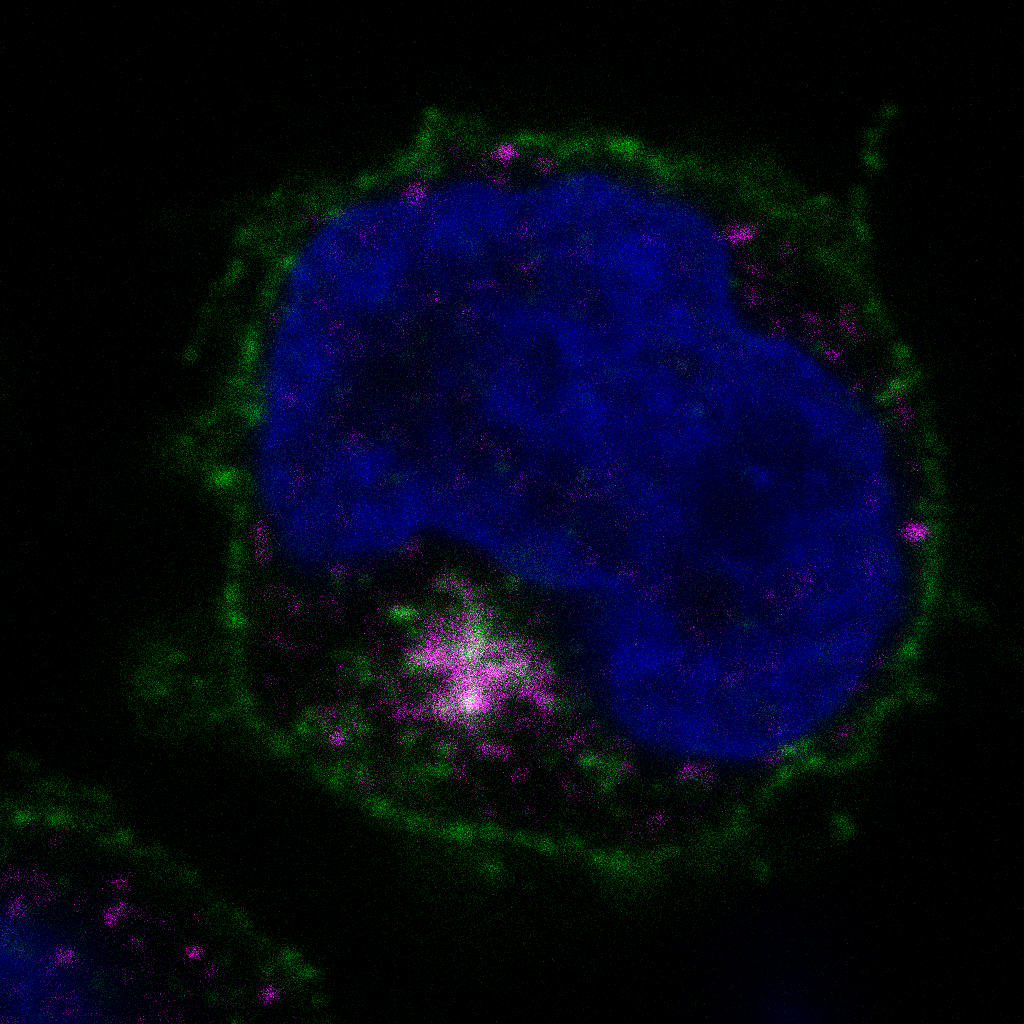

Supplement: Supplementary file 3 — Source data Fig. 1 [file 44319_2024_254_MOESM3_ESM.zip › Source data for Fig. 1h/Fig. 1h/RAB11_Merge.tif]

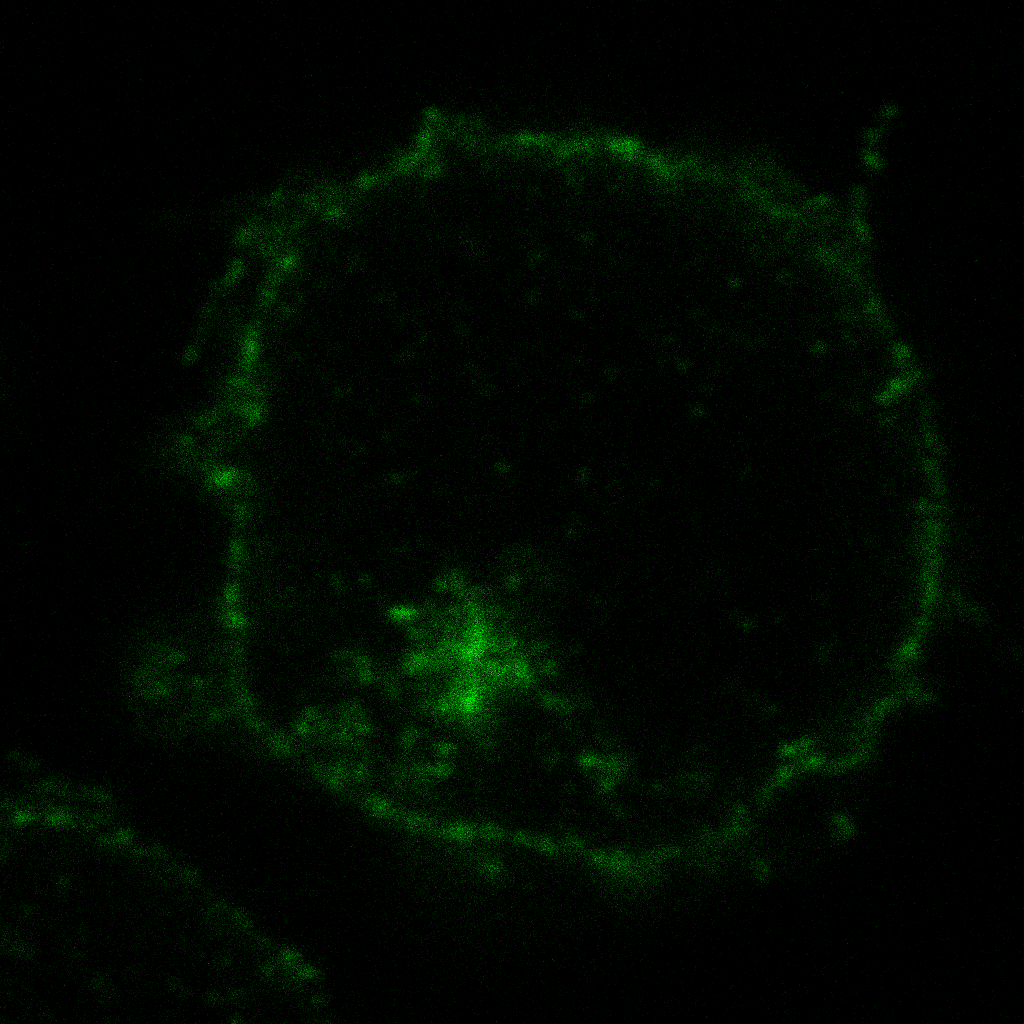

Supplement: Supplementary file 3 — Source data Fig. 1 [file 44319_2024_254_MOESM3_ESM.zip › Source data for Fig. 1h/Fig. 1h/RAB11_PD-1.tif]

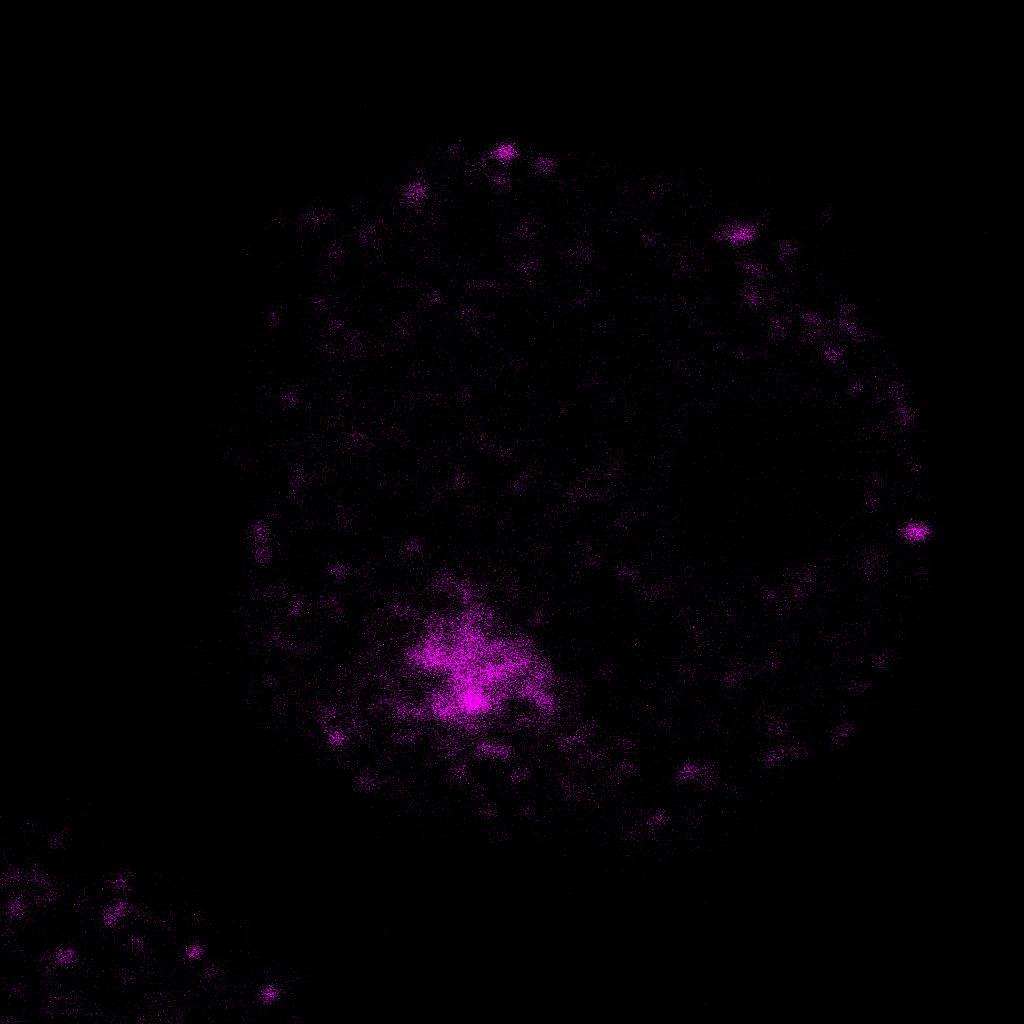

Supplement: Supplementary file 3 — Source data Fig. 1 [file 44319_2024_254_MOESM3_ESM.zip › Source data for Fig. 1h/Fig. 1h/RAB11_RAB11.tif]

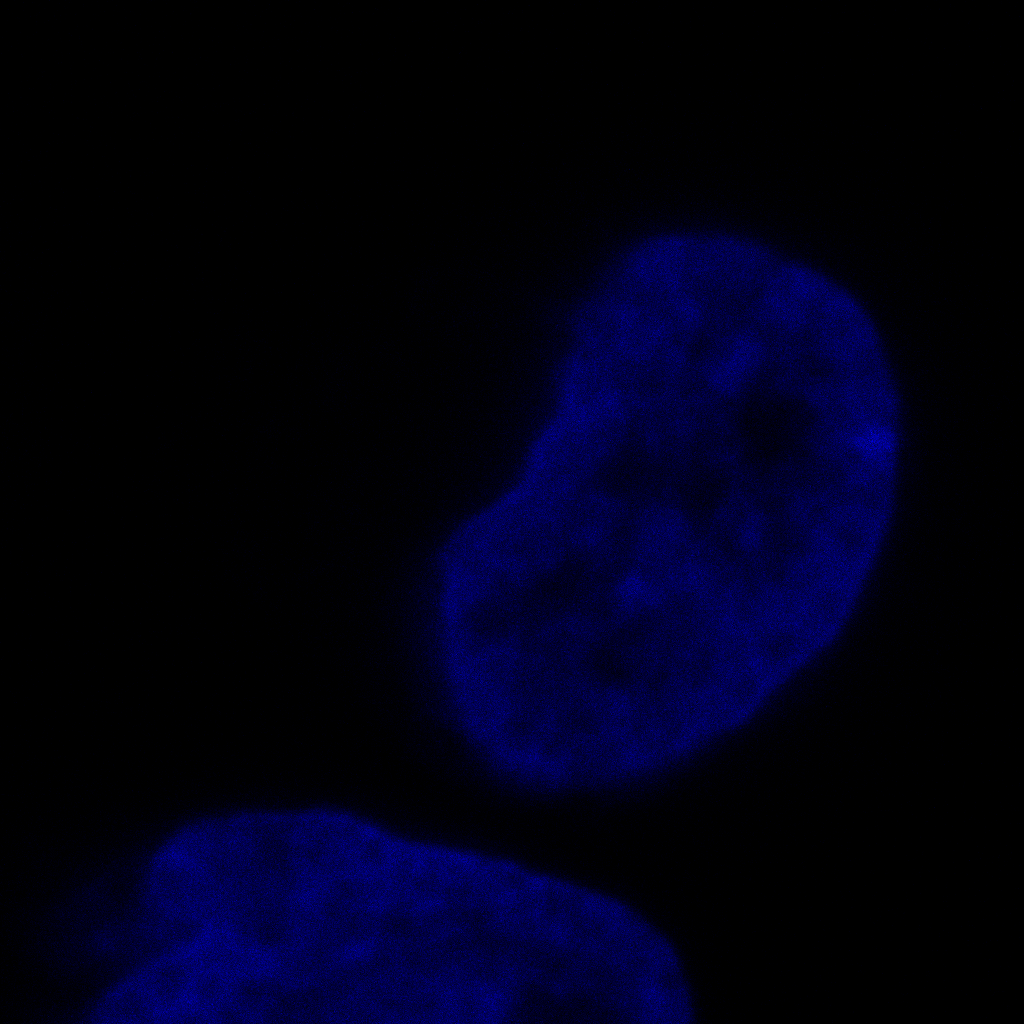

Supplement: Supplementary file 3 — Source data Fig. 1 [file 44319_2024_254_MOESM3_ESM.zip › Source data for Fig. 1h/Fig. 1h/TFR_DAPI.tif]

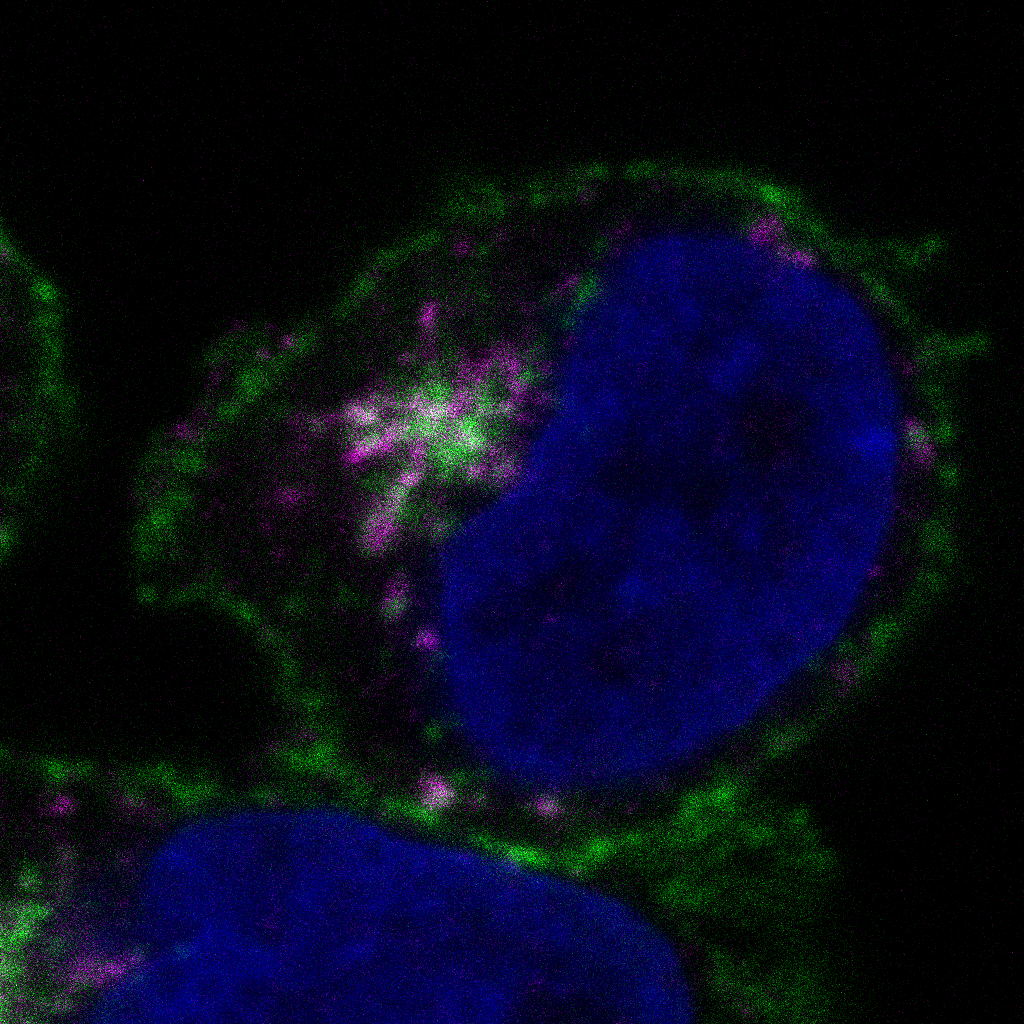

Supplement: Supplementary file 3 — Source data Fig. 1 [file 44319_2024_254_MOESM3_ESM.zip › Source data for Fig. 1h/Fig. 1h/TFR_Merge.tif]

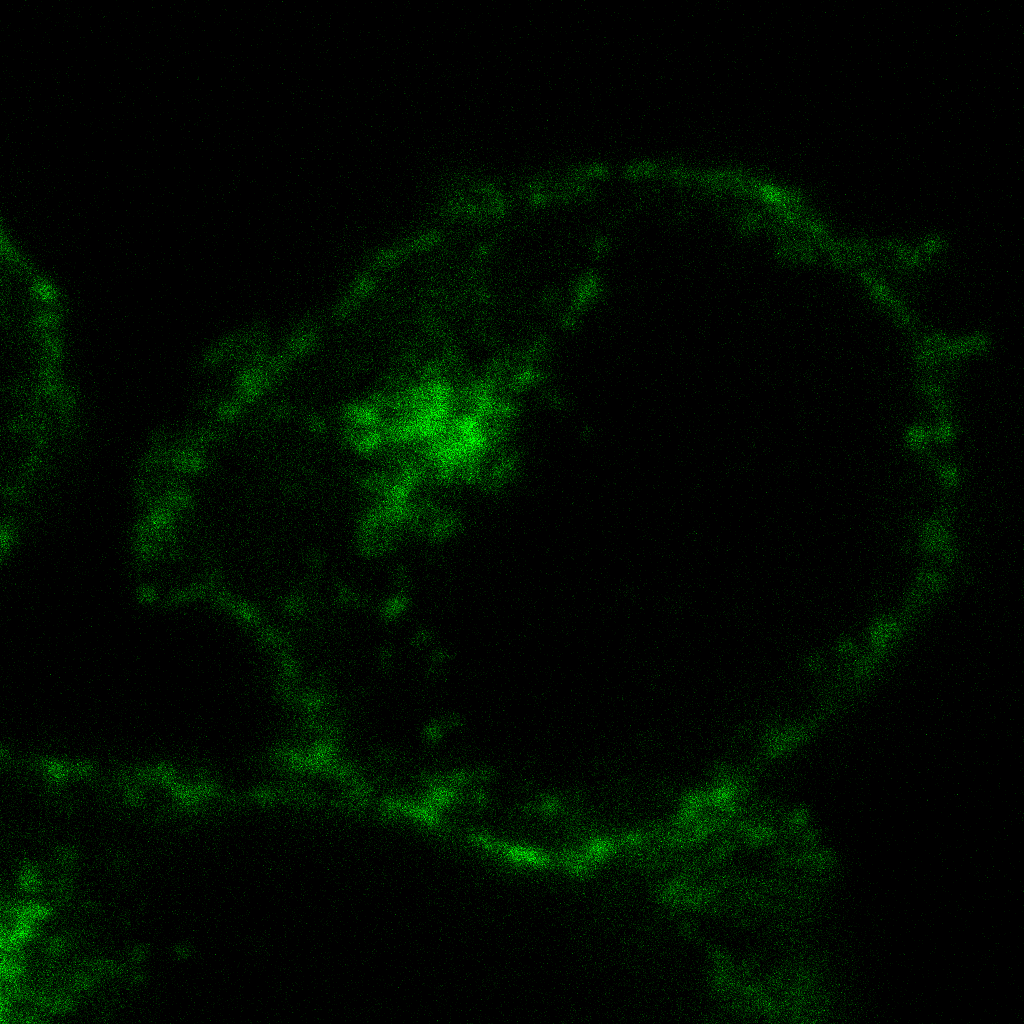

Supplement: Supplementary file 3 — Source data Fig. 1 [file 44319_2024_254_MOESM3_ESM.zip › Source data for Fig. 1h/Fig. 1h/TFR_PD-1.tif]

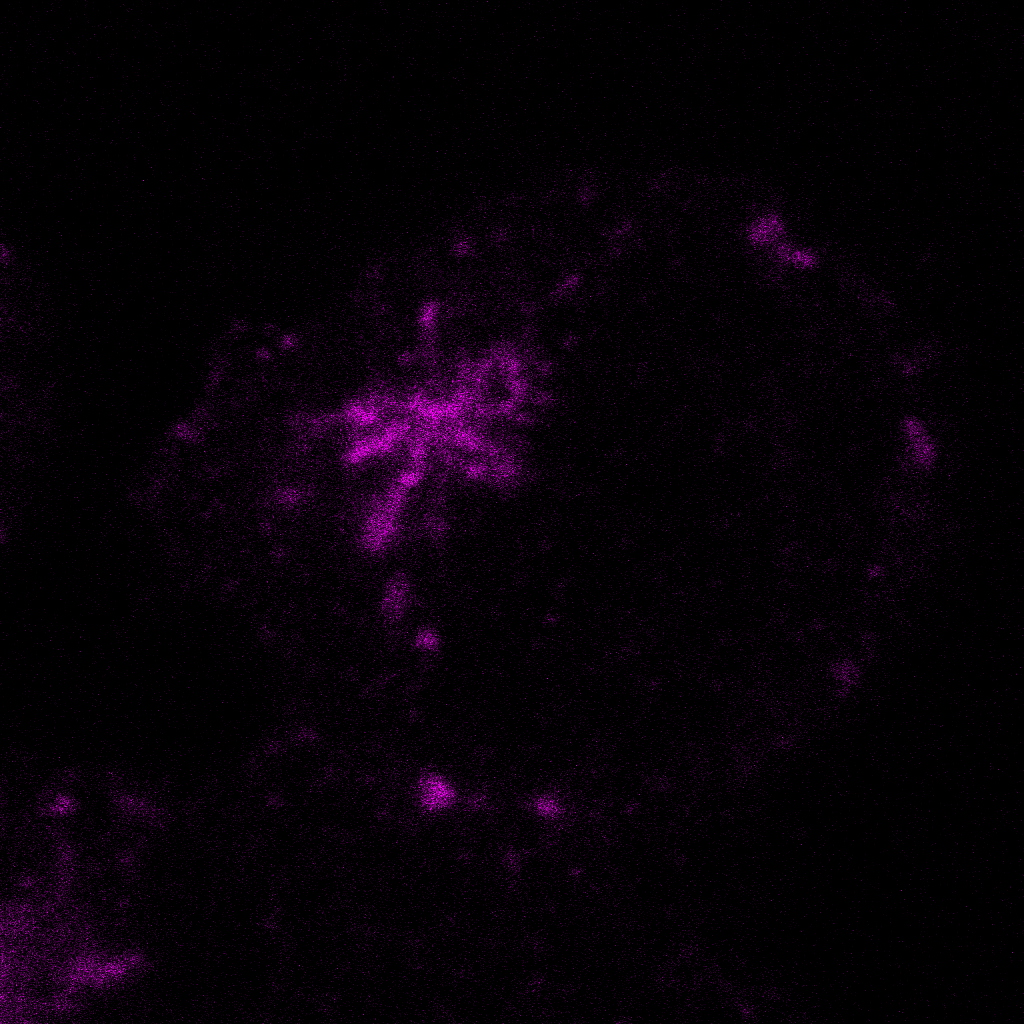

Supplement: Supplementary file 3 — Source data Fig. 1 [file 44319_2024_254_MOESM3_ESM.zip › Source data for Fig. 1h/Fig. 1h/TFR_TFR.tif]

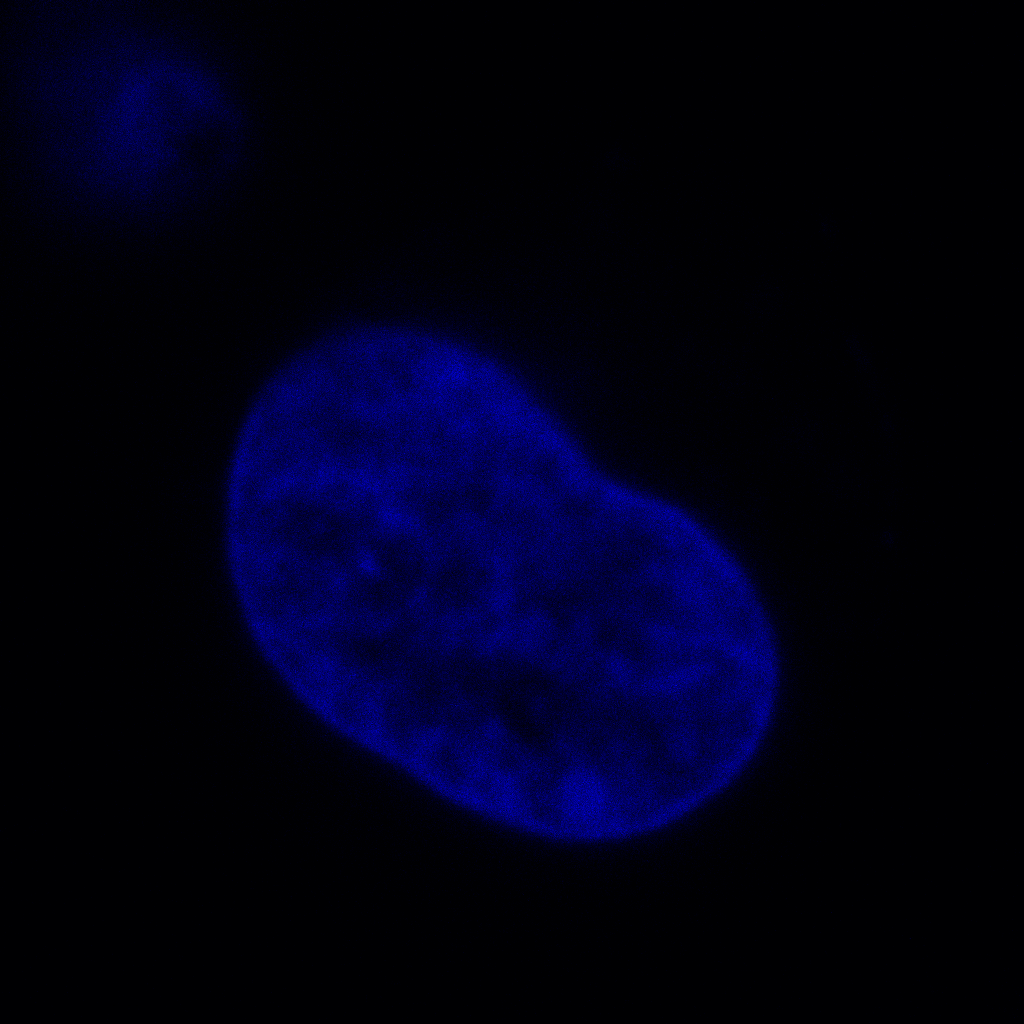

Supplement: Supplementary file 3 — Source data Fig. 1 [file 44319_2024_254_MOESM3_ESM.zip › Source data for Fig. 1h/Fig. 1h/TGN46_DAPI.tif]

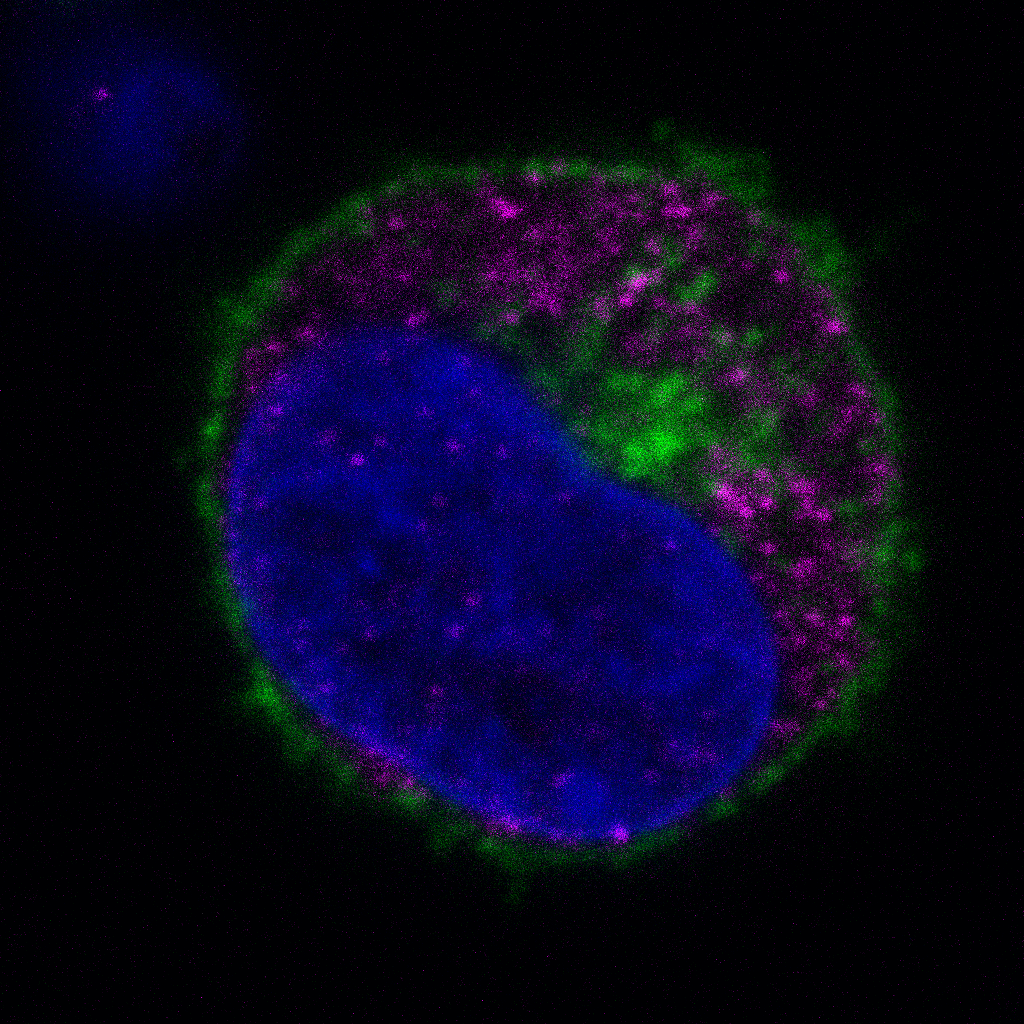

Supplement: Supplementary file 3 — Source data Fig. 1 [file 44319_2024_254_MOESM3_ESM.zip › Source data for Fig. 1h/Fig. 1h/TGN46_Merge.tif]

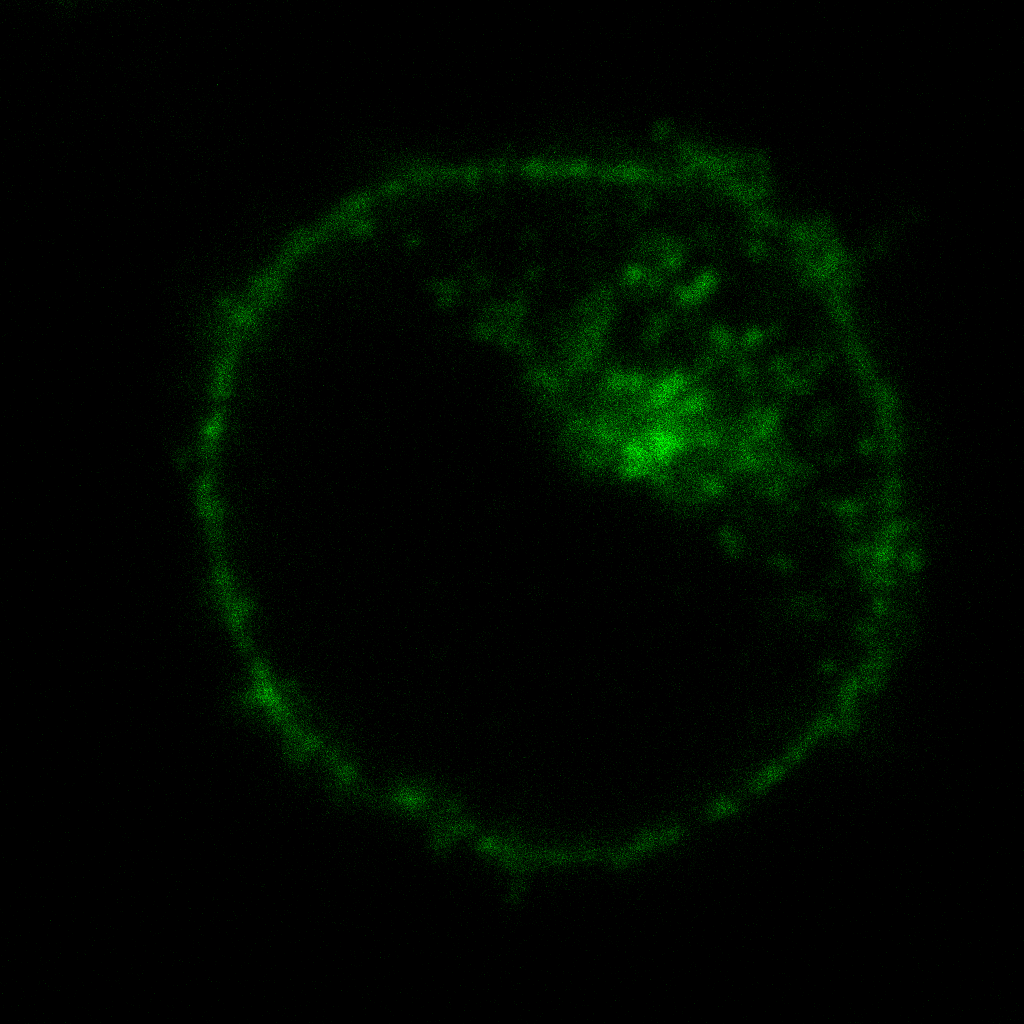

Supplement: Supplementary file 3 — Source data Fig. 1 [file 44319_2024_254_MOESM3_ESM.zip › Source data for Fig. 1h/Fig. 1h/TGN46_PD-1.tif]

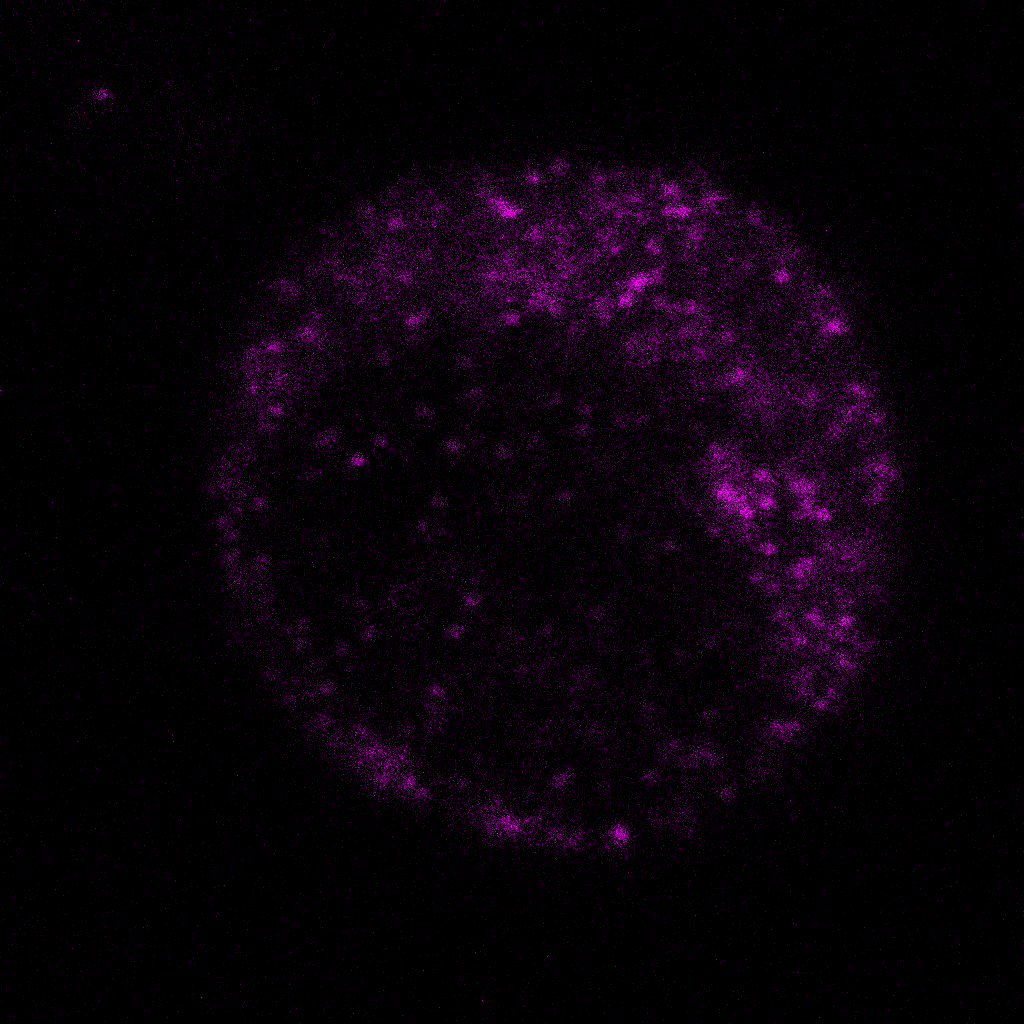

Supplement: Supplementary file 3 — Source data Fig. 1 [file 44319_2024_254_MOESM3_ESM.zip › Source data for Fig. 1h/Fig. 1h/TGN46_TGN46.tif]
